# Genome-Wide Identification of the Transcription Factors Involved in *Citrus* Fruit Ripening from the Transcriptomes of a Late-Ripening Sweet Orange Mutant and Its Wild Type

**DOI:** 10.1371/journal.pone.0154330

**Published:** 2016-04-22

**Authors:** Juxun Wu, Lili Fu, Hualin Yi

**Affiliations:** Key Laboratory of Horticultural Plant Biology (Ministry of Education), College of Horticulture and Forestry Science, Huazhong Agricultural University, Wuhan, 430070, China; Key Laboratory of Horticultural Plant Biology (MOE), CHINA

## Abstract

Fruit ripening is a genetically programmed process. Transcription factors (TFs) play key roles in plant development and ripening by temporarily and spatially regulating the transcription of their target genes. In this study, a total of 159 TFs were identified from a spontaneous late-ripening mutant 'Fengwan' (*C*. *sinensis* L. Osbeck) sweet orange (MT) and its wild-type counterpart ('Fengjie 72–1', WT) along the ripening period via the Transcription Factor Prediction of PlantTFDB 3.0. Fifty-two differentially expressed TFs were identified between MT and WT; 92 and 120 differentially expressed TFs were identified in WT and MT, respectively. The Venn diagram analysis showed that 16 differentially expressed TFs were identified between MT and WT and during the ripening of WT and MT. These TFs were primarily assigned to the families of C2H2, Dof, bHLH, ERF, MYB, NAC and LBD. Particularly, the number of TFs of the ERF family was the greatest between MT and WT. According to the results of the WGCNA analysis, a weighted correlation network analysis tool, several important TFs correlated to abscisic acid (ABA), citric acid, fructose, glucose and sucrose were identified, such as *RD26*, *NTT*, *GATA7* and *MYB21/62/77*. Hierarchical cluster analysis and the expression analysis conducted at five fruit ripening stages further validated the pivotal TFs that potentially function during orange fruit development and ripening.

## Introduction

Transcription factors (TFs) play key roles in plant development and stress responses through the temporary and spatial regulation of the transcription of target genes [1]. Many fruits develop from carpels (true fruit) or other floral-associated tissues (false or accessory fruit). During fruit development and ripening, TFs act as pivotal regulators. Several classes of transcription factors have defined functions in *Arabidopsis* and tomato carpel and fruit tissues, including *HD-Zip*, *KNOX*, *HB*, *SBP*, *BHLH*, *RAVB3*, *YABBY* and *AP2/ERF* [2, 3]. Screens for such regulators of fresh fruit ripening are important, and additional players remain elusive.

TFs are typically classified into different families based on their DNA-binding domains (DBDs); generally, TFs belonging to the same family have similar functions. Recent studies have indicated that an increasing number of TFs have been identified as having functions during fruit development and ripening in climacteric and non-climacteric fruit. For example, the overexpression of *VvABF2*, a bZIP family transcription factor, in grape cells resulted in the up-regulation and/or modification of groups of genes associated with abscisic acid (ABA) responses and enhanced responses to ABA treatment and changes in the synthesis of phenolic compounds and cell wall softening [4]. Silencing of *PacMYBA*, an R2R3-MYB transcription factor from red-colored sweet cherry cv. Hong Deng (*Prunus avium* L.), resulted in sweet cherry fruit lacking red pigment [5]. Another R2R3-MYB transcription factor, *FaMYB10*, has been identified as playing a major role in the regulation of flavonoid/phenylpropanoid metabolism during ripening of strawberry fruit [6]. In tomatoes and bananas, NAC transcription factors, such as *NAC1*/*NAC2*, may be involved in fruit ripening via interactions with ethylene signal components [7, 8].

The AP2/ERF gene family encodes plant-specific transcription factors that respond to developmental and environmental stimuli, and many of these factors function downstream of the ethylene, biotic, and abiotic stress signaling pathways [9]. In tomatoes, *LeERF2* is an important regulator of ethylene biosynthesis [10], and *SlAP2a* and *SlERF6* are negative regulators of fruit ripening. The RNAi repression of *SlAP2a* and *SlERF6* results in fruits that over-produce ethylene, ripen early and modify carotenoid accumulation [11, 12].

Cys2/His2 (C2H2)-type zinc finger proteins (ZFPs) are one of the largest families of transcriptional regulators in plants, which are important components in the regulation of plant growth, development, hormone responses, and tolerance to biotic and abiotic stresses [13, 14]. Previous studies have shown that C2H2-type zinc finger protein ZFP36 is a key regulator involved in abscisic acid-induced antioxidant defense and oxidative stress tolerance in rice [15] and that *Arabidopsis* C2H2 proteins AZF1 and AZF2 function as transcriptional repressors involved in the expression of abscisic acid-repressive and auxin-inducible genes under abiotic stress conditions [16]. The bHLH family has also been implicated in a range of functions in plants, frequently in conjunction with MYBs; the MYB-bHLH-WD40/WDR (MBW) regulatory complex is involved in regulating the biosynthesis of anthocyanins, which are important for coloration during fruit ripening [17]. The highly conserved N-terminal DOF region of the plant-specific DNA-binding-with-one-finger (Dof) family TF acts as a DNA-binding domain and corresponds to a conserved DNA *cis*-element (A/T)AAAG or its complementary inverse sequence [18]. Numerous studies have shown that Dof transcription factors are involved in various biological processes during plant growth and development, such as carbon and nitrogen metabolism, which can influence sugar accumulation in fruit [19], the light response, which is a significant regulatory factor for fruit ripening [20], flower and pollen development [21], and seed germination and development [22].

Citrus is one of the most important fruit crops worldwide and has a non-climacteric fruit maturation character [23]. During the ripening process of citrus, the expressions of a large number of genes are changed, up-regulated or down-regulated [24]. As transcriptional expression regulators, TFs play pivotal roles in this process. Recently, we examined 'Fengjie 72–1' and 'Fengwan' during the ripening period at the transcriptomic level [24]. 'Fengwan' sweet orange (MT) is a spontaneous late-ripening mutant from the ‘Fengjie 72–1’ orange (*Citrus sinensis* L. Osbeck) (WT) [24]. The mechanisms involved in the ripening of citrus fruit remain unclear, and only a few regulators have been reported. 'Fengjie 72–1' and 'Fengwan' have provided a promising platform to reveal the transcription factors involved in citrus fruit development and ripening. In this study, we created a protein sequence database of differential expression genes (DEGs) including the DEGs between MT and WT and DEGs of MT and WT during fruit ripening. This database was used to identify TFs in the Plant Transcription Factor Database v3.0 (PlantTFDB 3.0) [1]. Numerous TFs were identified, and we employed coexpression network analyses using the R package WGCNA [25] and qRT-PCR to identify the most credible and relevant TFs for citrus fruit ripening.

## Materials and Methods

### Plant materials and RNA preparation

Fruit samples of ‘Fengjie 72–1’ orange (*C*. *sinensis* L. Osbeck) (WT) and its spontaneous late-ripening mutant ‘Fengwan’ (MT), which were both cultivated in the same orchard (N31°03'35", E109°35'25") (Fengjie, Chongqing City, China), were harvested at 150, 170, 190, 210, and 240 d after flowering (DAF). Twelve representative fruits were sampled from each tree at each developmental stage. After separating the pulp from the peel, the pulp was sliced. The sliced WT pulp samples were combined (as for the MT samples), rapidly frozen in liquid nitrogen and stored at -80°C [26, 27]. A portion of the samples was used for extracting total RNA, as described previously [28]. Another aliquot was used for the determination of ABA, sugar and organic acid composition and concentration.

### Transcription factors isolation, identification and analysis

The WT and MT fruit pulps harvested at 170, 190 and 210 DAF were subjected to RNA-seq using an Illumina HiSeq^™^2000 at the Beijing Genomics Institute (Shenzhen). The RNA-seq data of these six fruit pulp samples of MT and WT, obtained in a previous study [24], were used in the present study, and the data of RNA-seq were submitted to the Gene Expression Omnibus (www.ncbi.nlm.nih.gov/geo/), accession number GSE69432. The gene expression levels were calculated using the RPKM (Reads Per kb per Million reads) method according to Zheng *et al*. [29]. Referring to the previous studies [29, 30], the Poisson model provides a natural framework for identifying differentially expressed genes. Denoting the number of unambiguous clean reads from a given gene as x, and considering that the expression of every gene occupies only a small part of the library, p(x) would closely follow the Poisson distribution,$P(x)\;=\;\frac{e^{-\lambda }\lambda^{x}}{x!}$ (λ is the real transcripts of the gene). A strict algorithm was used to identify differentially expressed genes between the two samples. The total clean read number of sample 1 is N1, and the total clean read number of sample 2 is N2; and gene A holds x reads in sample1 and y reads in sample2. The probability of gene A expressed equally between the two samples can be calculated as $2\sum_{i\;=\;0}^{i-y}p(i|x)$ or $2(1-\sum_{i\;=\;0}^{i-y}p(i|x))$(if $\sum_{i\;=\;0}^{i-y}p(i|x)>0.5),P(yx)\;=\;{\left(\frac{N2}{N2}\right)}^{y}\frac{(x+y)!}{x!y!{\left(1+\frac{N2}{N1}\right)}^{x+y+1}}$. The p-value corresponds to the differential gene expression test. FDR (False Discovery Rate) is a method used to determine the threshold of P-value in multiple tests [31]. ‘FDR ≤ 0.001 and the absolute value of log_2_Ratio ≥ 1’ was used as the thresholds to judge the significance of differences in gene expression. The values of fold-change with their respective P-values and FDR values for all genes were listed in S1 Table. A total of 18879 genes of WT and MT (S2 Table), 628 differential expression genes (DEGs) between MT and WT, 1036 DEGs between different ripening stages in WT and 1406 DEGs between different ripening stages in MT were used as original databases for transcription factor identification [24]. The protein sequences of these genes were isolated from the citrus genome (http://citrus.hzau.edu.cn/). The protein sequences of identified TFs were aligned against the GO database and KEGG pathway database using KOBAS 2.0 (http://kobas.cbi.pku.edu.cn/) [32] to perform enrichment analysis. The corrected P-value < 0.01 was set as cutoff for enrichment. REVIGO [33] was used to visualize and summarize the terms corresponding to biological processes and molecular functions identified using KOBAS 2.0.

The *Arabidopsis* TFs database of PlantTFDB 3.0 [1] was used as the reference TF database. The Transcription Factor Prediction algorithm, in which HMMER 3.0 [34] was used to identify TFs and assign these genes to different families [1], was performed to identify TFs. The best BLAST hits had maximal e-values of 1e-10. A cluster analysis was performed on the TF cluster of MT vs WT according to Eisen et al. [35] using Cluster 3.0. The log_2_ of RKPM for each TF was used for hierarchical clustering analysis.

### Gene Network Construction

The WGCNA (v1.42) package in R was used to construct coexpression networks [25]. A total of 18879 genes (S2 Table) with RKPM higher than 0.3 were used for WGCNA unsigned coexpression network analysis. The modules were obtained using the automatic network construction function blockwiseModules with default settings, except that the maxBlockSize was 19000, the TOMType was unsigned, the minModuleSize was 30, and the mergeCutHeight was 0.25. Once the network modules were identified, we validated their membership using a permutation procedure according to a previous study [36]. When the modules truly showed statistical and potentially functional relevance, the average TO (topological overlap) should be higher than that of random groups of genes of similar size. The eigengene value was calculated for each module and used to test the association with each sample. The total connectivity and intramodular connectivity (function softConnectivity), kME (for modular membership, also known as eigengene-based connectivity), and kME-P value were calculated for the 18879 genes clustered into 32 modules. The module eigengenes to relate consensus modules to physiological data and the 16 TFs identified in all three clusters DEGs of MT, WT and MT vs WT were also performed via WGCNA. These physiological data were measured in a previous study [24], which included malic acid, citric acid, quinic acid, fructose, glucose, sucrose and abscisic acid (ABA). In the present study, we used the RPKM of these 16 TFs and the physiological data of three ripening stages (170, 190 and 210 DAF) of WT and MT for the WGCNA analysis. A correlation coefficient (the absolute value) of more than 0.8 and p-value < 0.05 was used as the cutoff criteria for identifying the significance between physiological traits/TFs and modules.

### RNA Isolation and real-time quantitative PCR analysis

Total RNA were extracted from the samples of MT and WT harvested at 150, 170, 190, 210, and 240 DAF, as previously described [37]. The sequences of the primer pairs designed using Primer Express 3.0 (Applied Biosystems, Foster City, CA, USA) listed in S3 Table. The qRT-PCR analysis was conducted using an ABI 7900HT Fast Real-time system (Applied Biosystems) with the *GAPDH* gene as the reference [38], as previously described [24]. Real-time PCR was conducted with three replicates for each sample, and the data are indicated as the means ± standard error (SE) (n = 3).

## Results

### Identification of differentially expressed transcription factors during citrus fruit ripening

In a previous study [24], the transcriptomes of fruit pulps of MT and WT at the ripening stages 170, 190 and 210 DAF were analyzed. In the present study, a total of 18879 genes in these six transcriptomes were used to identify TFs (S2 Table). A total of 934 TFs were identified in WT and MT, 922 TFs were identified in MT and 929 TFs were identified in WT (S4 Table). These 934 TFs were assigned to 57 different families, the top three families of which were bHLH (71 TFs), NAC (64 TFs) and ERF (58 TFs) (S1 Fig).

**Fig 1:**
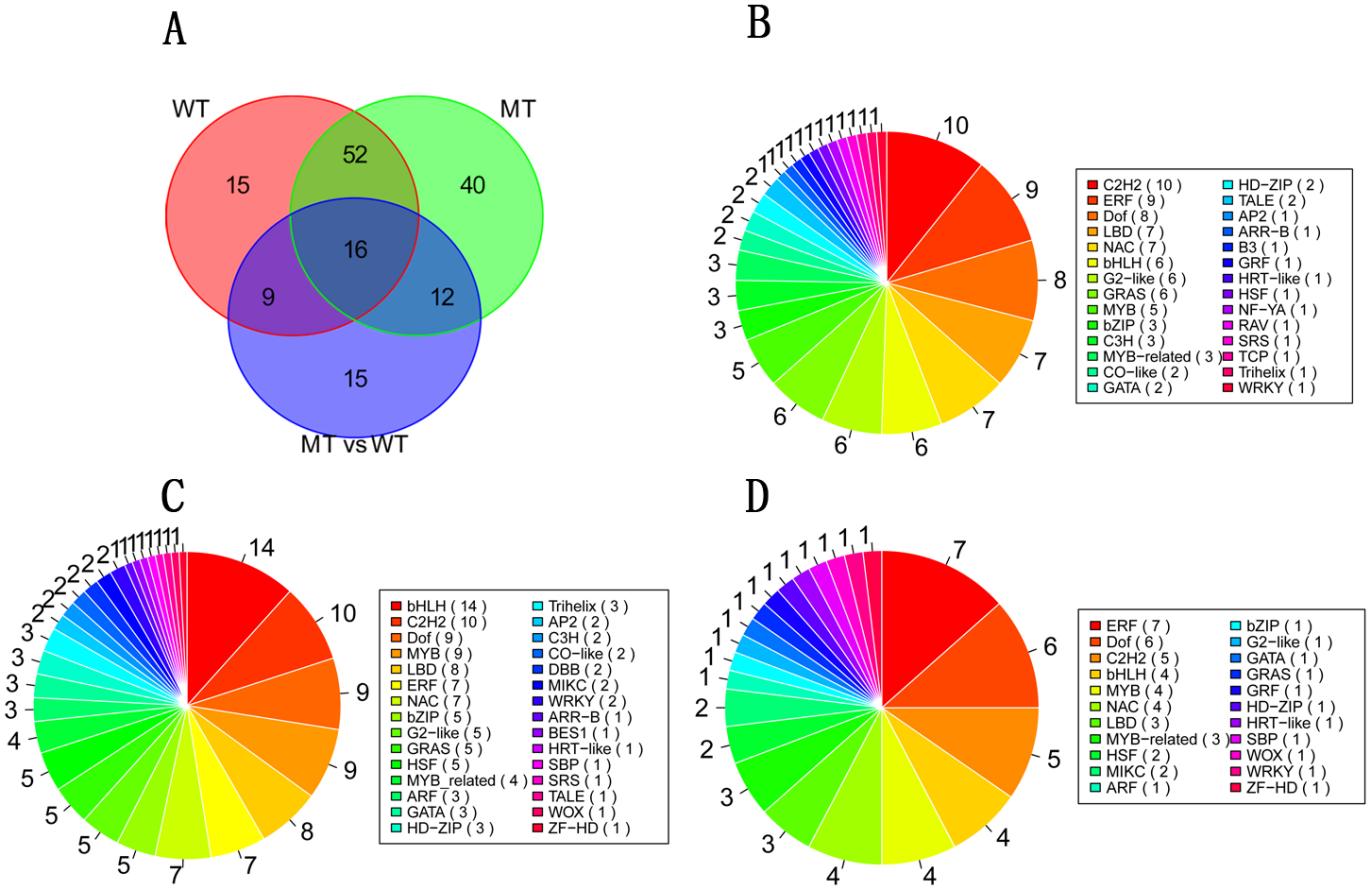
The Venn Diagram analysis (A) and the families assignment of TFs of WT (B), MT (C) and MT vs WT (D). MT vs WT indicate the TF cluster, which is differentially expressed between MT and WT.

We used a stringent value of FDR ≤ 0.001 and *P* value < 0.05 as the threshold to judge the significant differences in the gene expressions. A total of 1036 and 1406 genes differently expressed (≥ 2-fold) in WT and MT during fruit ripening, respectively. The protein sequences of these two cluster DEGs were used as the original database for transcription factor searching. The *Arabidopsis* TF database of PlantTFDB 3.0 [1] was used as the reference TF database. The Transcription Factor Prediction algorithm [1] was performed to identify TFs. A total of 144 TFs were identified including 92 TFs in the DEG cluster of WT and 120 TFs in the DEG cluster of MT (S5 Table; Fig 1A). According to the Venn diagram analysis, 68 TFs were identified in both WT and MT DEG clusters (Fig 1A; S5 Table).

As shown in Fig 1B and 1C, TFs were assigned to different families: 28 families in WT and 30 families in MT. The top three families of WT, containing the greatest number of TFs, were C2H2 (10 TFs), ERF (9) and Dof (8) (Fig 1B), and the top three families of MT were bHLH (14), C2H2 (10), Dof (9) and MYB (9) (Fig 1C). Notably, the C2H2 and Dof families were consistently in the top three families in both WT and MT.

### Function analysis of TFs identified in both MT and WT during fruit ripening

**Fig 2:**
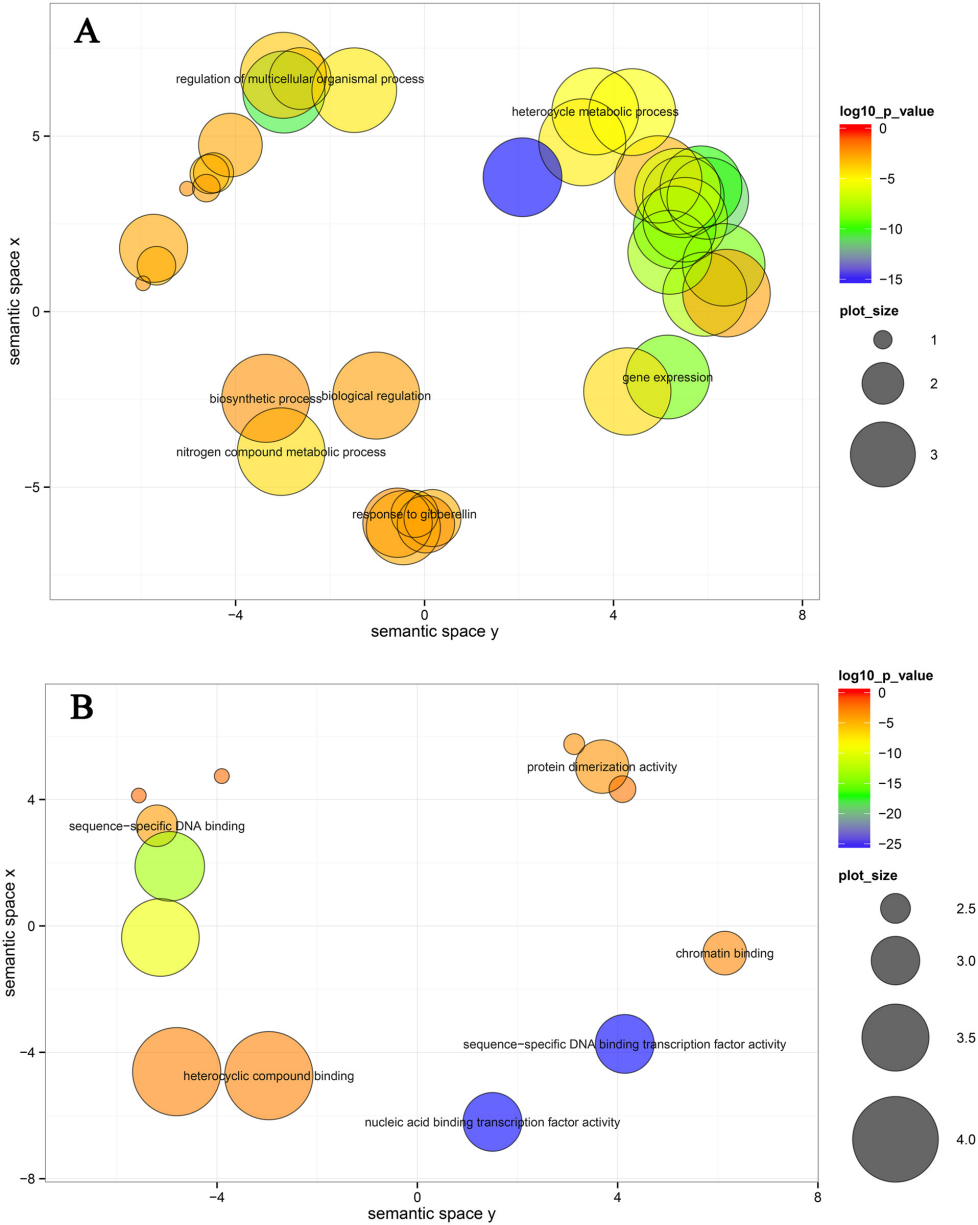
Biological process (A) and molecular function (B) enrichment analysis of the TFs differentially expressed during fruit ripening in both MT and WT. Bubble color indicates the p-value; plot size indicates the frequency of the GO term in the underlying GOA database (bubbles of more general terms are larger).

To gain a better understanding of the role of TFs in fruit ripening, GO-based term classification and KEGG-based pathway enrichment were performed. Using a cutoff of corrected P-value < 0.01, 68 TFs, which were differentially expressed in both MT and WT during fruit ripening, were enriched to 37 biological processes and 14 molecular functions after summarizing the GO terms by removing redundant GO terms using REViGO [33] (S6 Table). In biological processes, several hubs, including response to gibberellin, gene expression, regulation of multicellular organismal process, biological regulation, heterocycle metabolic process, nitrogen compound metabolic process and biosynthetic process, were significantly enriched (Fig 2A). Nucleic acid binding transcription factor activity, sequence-specific DNA binding transcription factor activity, sequence-specific DNA binding, protein dimerization activity, chromatin binding, and heterocyclic compound binding were significantly enriched in molecular function (Fig 2B). However, there was only one enrichment KEGG pathway to been identified (data not shown). One GRAS family transcription factor *GAI* (Cs2g16940) and one ARR-B family gene *ARR12* (Cs7g06180) were enriched in the plant hormone signal transduction pathway.

Plant hormones are important for fruit development and ripening. In the present study, 8, 9 and 10 TFs were enriched in the biological processes of ‘response to gibberellin’, ‘response to salicylic acid’ and ‘response to ethylene’, respectively (S6 and S7 Tables). Some TFs were enriched in different biological processes, for example, *GAI* (Cs2g16940) was enriched in ‘response to gibberellin’, ‘response to salicylic acid’ and ‘response to ethylene’ and *MYB77* (Cs3g23950) was enriched in ‘response to salicylic acid’ and ‘response to ethylene’ (S7 Table).

### Differentially expressed transcription factors between MT and WT

In a previous study [24], a total of 628 genes were differently expressed (≥ 2-fold) between MT and WT. The protein sequences of this cluster DEGs were used as the original database for transcription factor searching. A total of 52 differentially expressed TFs between MT and WT were identified, the TF cluster MT vs WT (Fig 1A and Table 1). MT is a later-ripening bud mutant of WT; therefore, the extensive analysis of these 52 TFs will identify important TFs involved in later-ripening trait formation.

**Table 1:** Differential expression transcription factors (TFs) between MT and WT. 170, 190 and 210 indicate 170, 190 and 210 DAF, respectively. The change fold is shown as a log_2_ ratio. Module colors were obtained from the analysis of WGCNA. Clusters were obtained from hierarchical clustering analysis via Cluster 3.0.

GeneID	Family	Module color	Cluster	Fold change (MT/WT)			Description
				170	190	210	
Cs9g16810	ERF	lightyellow	I	-0.18	0.82	1.76	C-repeat-binding factor 4
Cs2g05620	ERF	red	I	1.18	0.54	1.49	ERF domain protein 9
Cs1g07950	ERF	blue	I	2.22	0.66	0.91	ethylene responsive element binding factor 4
Cs3g19420	ERF	midnightblue	I	1.05	0.92	1.50	Integrase-type DNA-binding superfamily protein
Cs1g11880	ERF	lightgreen	II	-1.40	-0.32	-0.12	Integrase-type DNA-binding superfamily protein
Cs9g13610	ERF	black	II	0.09	-1.12	-0.25	Integrase-type DNA-binding superfamily protein
Cs5g29870	ERF	blue	II	-2.47	0.57	0.37	ethylene response factor 1
Cs1g23230	Dof	turquoise	I	1.05	1.01	0.75	OBF binding protein 1
Cs7g03670	Dof	red	I	1.08	-0.42	0.98	cycling DOF factor 2
orange1.1t01261	Dof	purple	II	-1.22	-1.19	-0.10	Dof-type zinc finger DNA-binding family protein
Cs5g01740	Dof	brown	II	-0.08	-1.07	-0.83	Dof-type zinc finger DNA-binding family protein
Cs3g21070	Dof	lightgreen	II	-3.19	-0.26	-0.39	Dof-type zinc finger DNA-binding family protein
Cs8g18320	Dof	brown	IV	-0.32	-2.13	-10.36	Dof-type zinc finger DNA-binding family protein
Cs8g17960	C2H2	cyan	I	1.04	0.26	0.65	C2H2-type zinc finger family protein
Cs7g01850	C2H2	turquoise	I	0.57	1.03	0.75	C2H2-type zinc finger family protein
Cs8g04280	C2H2	yellow	II	-1.23	-0.28	0.15	salt tolerance zinc finger
Cs3g02080	C2H2	blue	II	-0.48	-1.11	-0.33	indeterminate(ID)-domain 5
Cs7g21900	C2H2	red	III	-7.18	-2.94	-2.64	C2H2-type zinc finger family protein
Cs7g19870	bHLH	turquoise	II	-1.27	-0.15	-0.08	bHLH DNA-binding superfamily protein
Cs9g13930	bHLH	turquoise	II	-0.58	-1.01	-0.23	bHLH DNA-binding superfamily protein
Cs1g02580	bHLH	tan	II	-1.08	-0.17	-0.05	bHLH DNA-binding superfamily protein
Cs6g21120	bHLH	yellow	VI	0.05	10.97	-7.16	bHLH DNA-binding family protein
Cs6g21530	MYB	turquoise	I	1.06	0.66	-0.35	myb domain protein 16
Cs2g12700	MYB	green	II	-2.41	0.38	0.00	myb domain protein 62
Cs6g01750	MYB	cyan	II	-2.72	-1.37	-0.68	myb domain protein 61
Cs3g23950	MYB	yellow	V	0.06	2.07	9.66	myb domain protein 77
Cs8g02020	MYB_related	saddlebrown	II	-0.71	-0.63	-1.19	myb-like HTH transcriptional regulator family protein
Cs7g31610	MYB_related	turquoise	II	-1.09	0.26	0.15	Duplicated homeodomain-like superfamily protein
Cs2g27940	MYB_related	red	III	-6.21	-2.84	-2.18	myb domain protein 21
Cs8g14700	NAC	red	I	1.74	1.49	1.88	NAC domain containing protein 61
Cs1g06760	NAC	yellow	II	-1.23	-0.38	-0.05	NAC (No Apical Meristem) domain transcriptional regulator superfamily protein
Cs5g29650	NAC	green	II	-1.40	-1.85	-0.23	NAC domain containing protein 74
Cs2g13920	NAC	brown	II	0.11	-1.94	-2.55	NAC domain containing protein 84
Cs8g04300	LBD	blue	II	-1.34	-1.66	0.42	LOB domain-containing protein 38
Cs7g30620	LBD	yellow	II	-3.12	0.12	0.20	Lateral organ boundaries (LOB) domain family protein
Cs7g26710	LBD	blue	II	-2.17	-0.03	-1.66	LOB domain-containing protein 41
Cs8g15030	bZIP	lightgreen	II	-1.90	-0.10	-0.13	bZIP transcription factor family protein
Cs5g32400	ARF	yellow	II	-1.27	-0.12	-0.37	auxin response factor 1
Cs5g26420	G2-like	brown	IV	0.05	-1.84	-8.70	Homeodomain-like superfamily protein
Cs5g26470	GATA	turquoise	I	1.22	1.06	1.04	GATA transcription factor 7
Cs1g23790	GRAS	turquoise	II	0.12	-1.27	1.26	GRAS family transcription factor
Cs6g15330	GRF	green	II	-0.25	-1.74	-0.24	growth-regulating factor 4
Cs1g23760	HD-ZIP	green	II	-2.95	-1.11	-0.67	homeobox protein 40
Cs4g13650	HRT-like	brown	II	0.24	-1.24	-0.64	effector of transcription2
Cs9g07650	HSF	turquoise	II	-1.30	0.09	-0.15	heat shock transcription factor A6B
Cs4g14590	HSF	yellow	II	-1.16	-0.36	-0.16	heat shock transcription factor A2
Cs7g11810	MIKC	purple	I	0.58	1.01	-0.11	K-box region and MADS-box transcription factor family protein
Cs5g17820	MIKC	blue	II	-2.15	-1.42	-0.69	K-box region and MADS-box transcription factor family protein
Cs7g10990	SBP	turquoise	I	1.31	1.09	0.95	Squamosa promoter-binding protein-like (SBP domain) transcription factor family protein
Cs3g23280	WOX	turquoise	I	3.54	1.38	0.00	WUSCHEL related homeobox 4
Cs2g02790	WRKY	yellow	II	-1.11	-0.32	0.02	WRKY family transcription factor
Cs6g21230	ZF-HD	turquoise	I	1.27	0.17	-0.12	mini zinc finger 2

**Fig 3:**
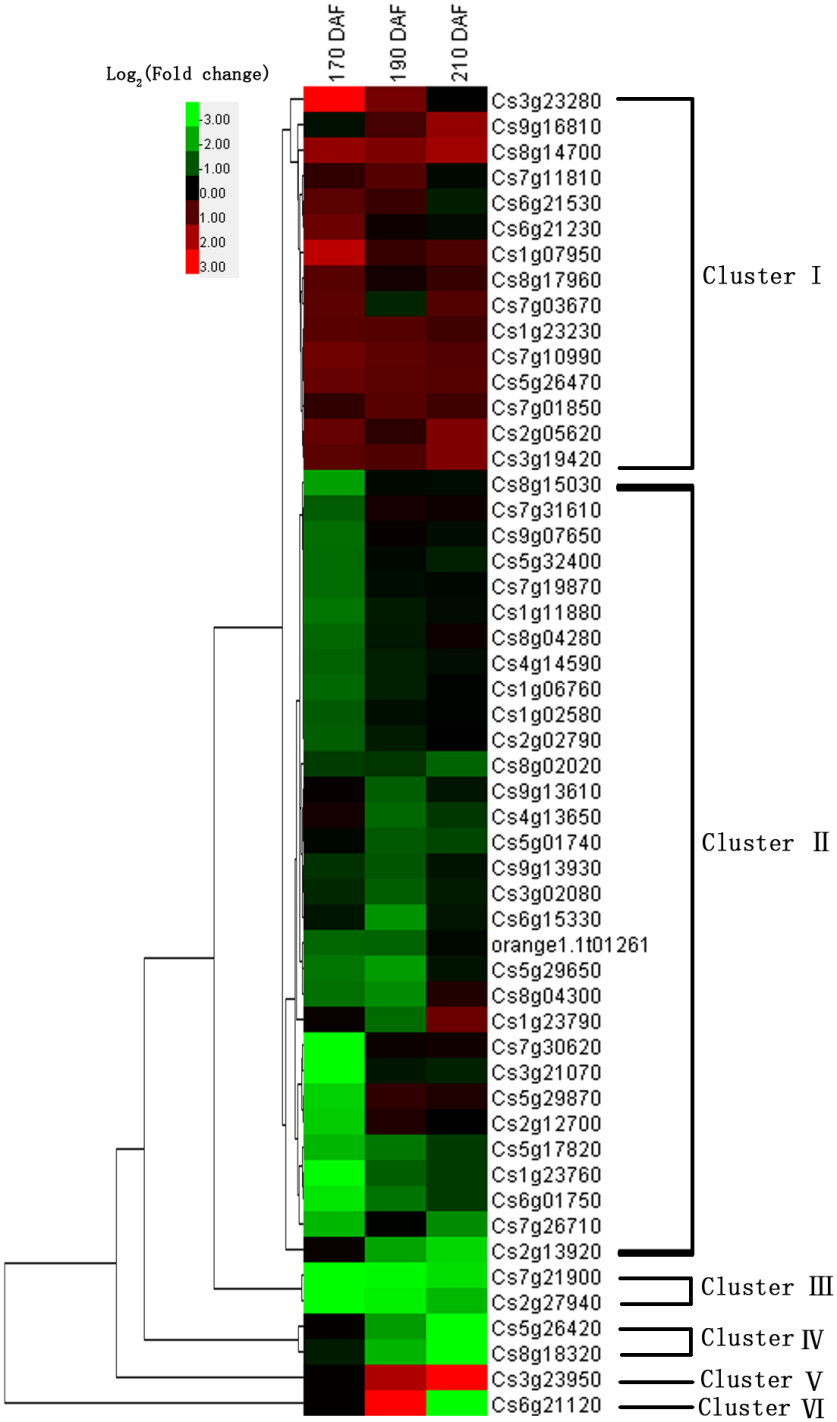
Hierarchical cluster analysis of the TF differential expressed between MT and WT.

As shown in Fig 1D, these 52 TFs were assigned to 22 different families. The top three families of the DEG cluster MT vs WT were ERF (7), Dof (6) and C2H2 (5). Thus, we focused on the ERF family TFs, as the ERF family contained the greatest number of TFs in the DEG cluster MT vs WT. The change fold of gene expression, the log_2_ ratio, between the MT and WT were performed with hierarchical cluster analysis using Cluster 3.0 (Fig 3). As shown in Fig 3, six clusters were identified. The TFs of cluster I were up-regulated in MT and TFs of cluster II were down-regulated in MT. The number of down-regulated TFs was much more than that of up-regulated TFs.

In cluster I, *NAC61* (Cs8g14700) and *GATA7* (Cs5g26470) were up-regulated more than 2-fold in MT at all three ripening stages; *ERF4* (Cs1g07950) and *WOX4* (Cs3g23280) were up-regulated more than 6-fold in MT at 170 DAF (Table 1). In cluster II, there were several TFs down-regulated more than 6-fold in MT, such as *MYB61/62* (Cs6g01750/Cs2g12700), Cs3g21070 (Dof family TF), *ERF1* (Cs5g29870) and *HB40* (Cs1g23760) (Table 1). Other clusters were TFs with a substantial change between MT and WT, such as *MYB21/77* (Cs2g27940/ Cs3g23950) and *OBP2* (Cs8g18320), which were down/up-regulated in the range of 0 to hundreds fold (Table 1).

After removing redundant GO terms, these 52 TFs were enriched to 28 biological processes and 9 molecular functions (P-value < 0.01) (S8 Table). According to the result of REViGO [33], in biological process, most of TFs were assigned to ‘regulation of transcription, DNA-templated’, ‘response to ethylene’ and ‘nitrogen compound metabolic process’ (S2A Fig); in molecular function, most of TFs were assigned to ‘nucleic acid binding transcription factor activity’, ‘chromatin binding’, ‘sequence-specific DNA binding transcription factor activity’, ‘transcription regulatory region DNA binding’ and ‘heterocyclic compound binding’ (dispensability < 0.15) (S2B Fig). Interestingly, some TFs were enriched in hormone-related processes, such as ‘response to ethylene’ (10 TFs), ‘response to jasmonic acid’ (10), ‘response to gibberellin’ (6), ‘response to auxin’ (9) and ‘ethylene-activated signaling pathway’ (7). These TFs involved in hormone related processes might be candidate regulators for the formation of later-ripening trait, which were listed in S9 Table. Thereinto, *MYB16* (Cs6g21530), *MYB21* (Cs2g27940) and *ERF4* (Cs1g07950) were assigned to different hormone response processes, indicating that these TFs might play a wide range of regulatory roles during citrus fruit ripening. In addition, three TFs were identified to enrich in plant hormone signal transduction pathway (data not shown). *ERF1* (Cs5g29870), *ARF1* (Cs5g32400) and *TGA9* (Cs8g15030) were assigned to ethylene, auxin and salicylic acid signal transduction pathways, respectively.

### Coexpression Network Analysis with WGCNA

**Fig 4:**
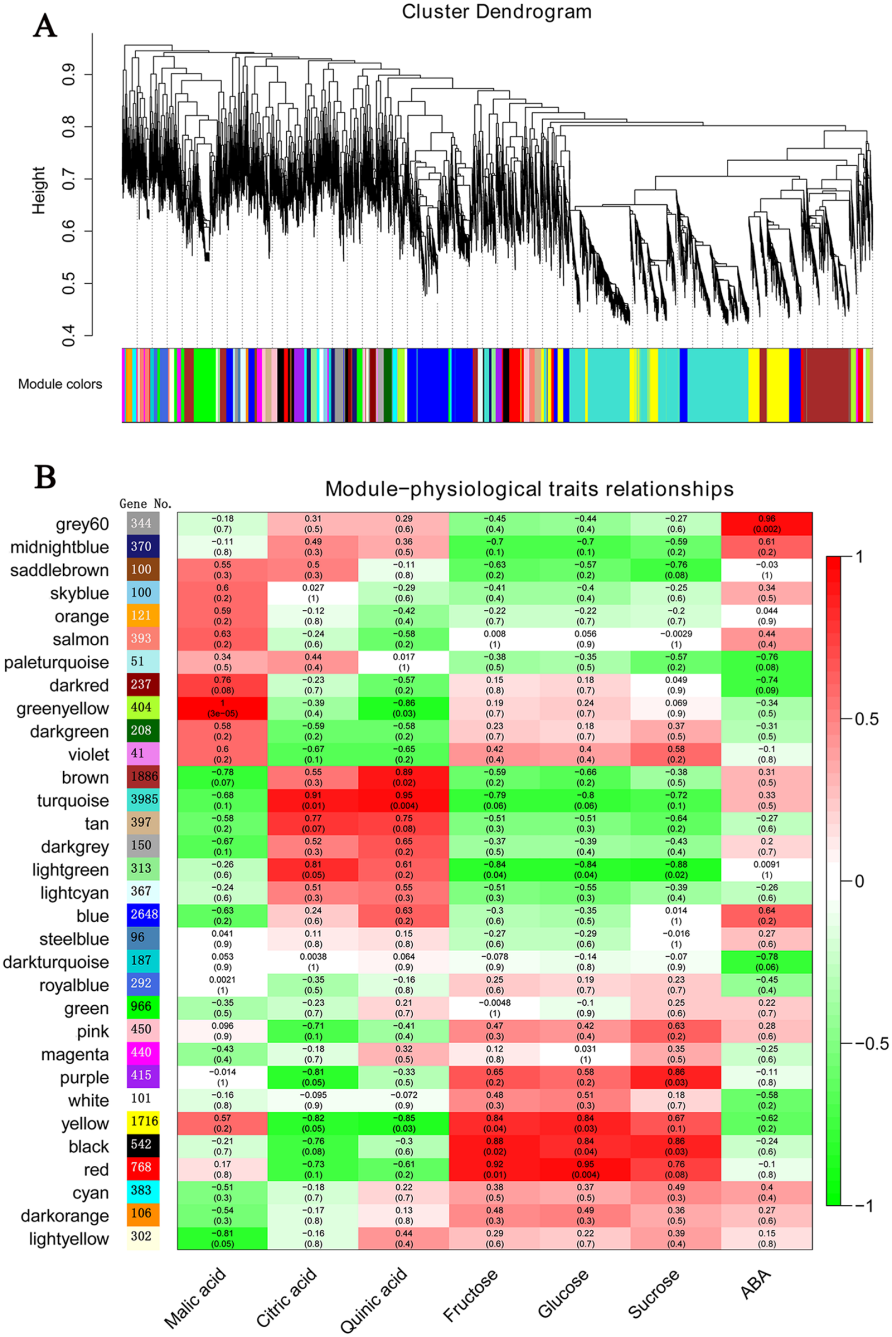
Hierarchical cluster tree with dissimilarity based on topological overlap showing coexpression modules identified by WGCNA (A). Each leaf in the tree is one gene. The major tree branches constitute 32 modules labeled by different colors. Module colors were determined in the single-block analysis. B, Module-physiological traits association. Each row corresponds to a module. The number of genes in each module is indicated on the left. Each column corresponds to a physiological trait. The color of each cell at the row-column intersection indicates the correlation coefficient between the module and the physiological trait, and the numbers in each cell indicate correlation coefficient R and P value, respectively.

TFs can regulate a large number of target genes, as these genes are characterized based on network regulation. Therefore, a weighted correlation network analysis tool, WGCNA, was adopted [25]. The WGCNA R software package is a systems biology approach whose purpose is to understand networks instead of individual genes. In the present study, coexpression networks were constructed based on pairwise correlations between the genes in common expression trends across all 18879 genes in all samples, including all three ripening stage transcriptomes of MT and WT (S2 Table). The modules are defined as clusters of highly interconnected genes, and genes within the same module are highly correlated with one another. The weighted correlation network analysis resulted in 32 distinct modules, labeled with different colors (Fig 4A). After validation using a permutation procedure according to a previous study [36], 24 modules displayed TO that was higher than what is expected for random groups of transcripts (S3 Fig); the modules of cyan, darkorange, darkturquoise, lightcyan, lightyellow, magenta, pink and royalblue had no truly statistical relevance. As shown in Fig 4A, each tree branch constitutes a module, and each leaf in the branch is one gene. Each module contained different numbers of genes. The turquoise module contained 3985 genes, which was the largest cluster of genes; the smallest module, violet module, only contained 41 genes (Fig 4B). The module eigengene is the first principal component of a given module and can be considered a representative of the gene expression profile of that module (S4 Fig). The TFs identified in the present study were assigned to different modules. As shown in S5 Fig, most TFs were assigned to turquoise, yellow, brown and blue modules. The turquoise module eigengene exhibited down-regulated expression during fruit ripening in WT and MT. In contrast, the yellow module eigengene was up-regulated expression during the fruit ripening of WT and MT. Interestingly, the expression patterns of brown and blue module eigengenes were different between WT and MT (S4 Fig).

In our previous study [24], we measured the content of soluble sugar, organic acid and abscisic acid (ABA) of WT and MT fruits at different ripening stages. These physiological trait data were used in the present study to perform a correlation network analyses with gene expression trends (Fig 4B). As shown in Fig 4B, malic acid was highly positively correlated with the greenyellow module (r = 1, p = 3e-05), and citric acid and quinic acid were all highly positively correlated with the turquoise module. For soluble sugars, fructose and glucose were all positively correlated with red, yellow and black modules, while sucrose was correlated with lightgreen, purple and black modules; ABA is a significant hormone for citrus fruit ripening, and in the present study, this hormone was highly positively correlated with the gray60 module.

**Fig 5:**
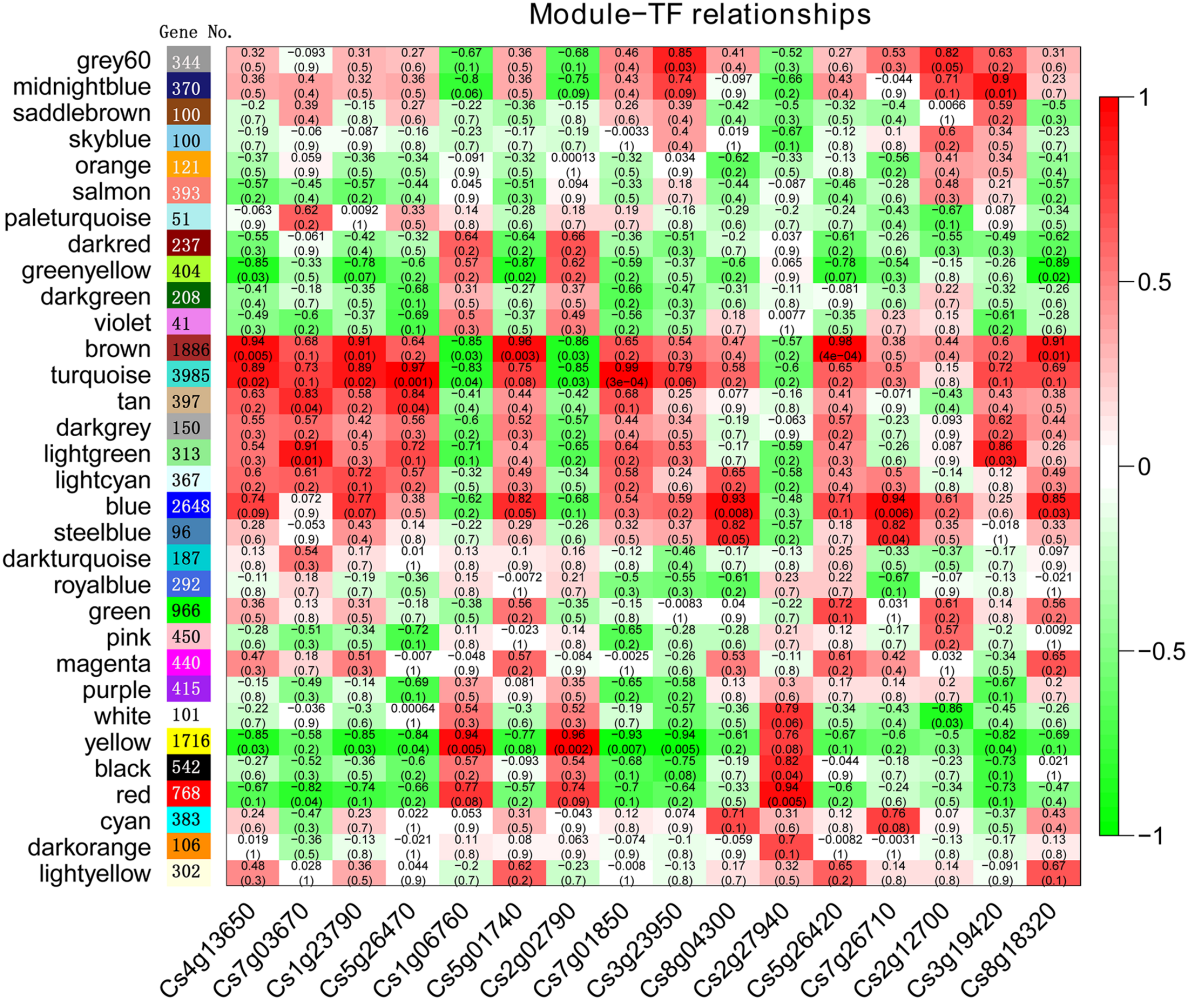
Module-TF association. Each row corresponds to a module. The number of genes in each module is indicated on the left. Each column corresponds to a TF. The color of each cell at the row-column intersection indicates the correlation coefficient between the module and the TF, and the numbers in each cell indicate correlation coefficient R and P value, respectively.

Sixteen TFs were identified in all three cluster DEGs, including MT, WT and MT vs WT (Fig 1A). These 16 TFs may play important roles in the citrus fruit ripening process. Thus, we conducted a correlation analysis between these 16 TFs and gene modules (Fig 5). As shown in Table 2 and Fig 5, *MYB77* (Cs3g23950) and *MYB62* (Cs2g12700) belonged to the yellow and green modules, respectively; however, these genes were all high positively correlated with the gray60 module, which was positively correlated with ABA. *RD26* (Cs1g06760) and *WRKY42* (Cs2g02790) belonged to the yellow module and also had the highest positive correlation with the yellow module, and *MYB21* (Cs2g27940) was highly positively correlated with the red module (Table 1 and Fig 5). These findings showed that *RD26*, *WRKY42* and *MYB21/77* had a high correlation with fructose and glucose. *HAM4* (Cs1g23790), *GATA7* (Cs5g26470) and *NTT* (Cs7g01850) belonged to the turquoise module, which was high positively correlated with citric acid and quinic acid (Table 2 and Fig 5). Additionally, 4 TFs, *ET2* (Cs4g13650), Dof 4.6 (Cs5g01740), *MYR2* (Cs5g26420) and *OBP2* (Cs8g18320), were highly positively correlated with the brown module, which was the largest cluster in these 16 TFs and had a positive correlation with quinic acid (Fig 5). Two Dof family TFs were in the brown module (Table 2 and Fig 5).

**Table 2:** Differential expression transcription factors (TFs) during fruit ripening of WT, MT and between MT and WT. 170, 190 and 210 indicate 170, 190 and 210 DAF, respectively. RPKM, reads per kb per million reads. E-value was calculated by BLAST.

Gene ID	Gene Name	Family	moduleColor	MT(RPKM)			WT(RPKM)			*A*. *thaliana* ortholog gene	E value
				170	190	210	170	190	210		
Cs1g06760	*RD26*	NAC	yellow	41.70	100.33	290.49	97.95	130.30	301.33	AT4G27410.2	1.00E-136
Cs2g02790	*WRKY42*	WRKY	yellow	3.05	9.34	26.03	6.56	11.66	25.68	AT4G04450.1	1.00E-166
Cs3g23950	*MYB77*	MYB	yellow	13.82	14.06	0.81	13.23	3.34	-	AT3G50060.1	5.00E-58
Cs1g23790	*HAM4*	GRAS	turquoise	9.43	2.54	1.95	8.70	6.11	0.82	AT4G36710.1	1.00E-162
Cs5g26470	*GATA7*	GATA	turquoise	33.37	7.76	2.76	14.32	3.73	1.34	AT4G36240.1	4.00E-36
Cs7g01850	*NTT*	C2H2	turquoise	25.55	10.73	4.68	17.24	5.25	2.77	AT3G57670.1	1.00E-125
Cs7g03670	*CDF2*	Dof	red	12.55	6.24	7.28	5.94	8.32	3.69	AT5G39660.2	1.00E-95
Cs2g27940	*MYB21*	MYB_related	red	-	1.02	7.00	0.07	7.32	31.64	AT3G27810.1	9.00E-55
Cs3g19420	*DREB26*	ERF	midnightblue	43.47	40.92	8.62	20.97	21.59	3.06	AT1G21910.1	4.00E-49
Cs2g12700	*MYB62*	MYB	green	0.80	4.80	-	4.25	3.69	-	AT1G68320.1	4.00E-97
Cs4g13650	*ET2*	HRT-like	brown	15.29	4.39	1.64	12.92	10.37	2.56	AT5G56780.1	1.00E-93
Cs5g01740	*Dof 4*.*6*	Dof	brown	12.95	6.02	2.66	13.67	12.64	4.72	AT4G24060.1	7.00E-73
Cs5g26420	*MYR2*	G2-like	brown	4.56	1.56	-	4.41	5.61	0.42	AT3G04030.3	1.00E-165
Cs8g18320	*OBP2*	Dof	brown	4.41	1.07	-	5.50	4.71	1.31	AT1G07640.2	3.00E-63
Cs8g04300	*LBD38*	LBD	blue	5.88	0.96	2.40	14.86	3.04	1.79	AT3G49940.1	7.00E-66
Cs7g26710	*LBD41*	LBD	blue	8.10	2.67	0.68	36.38	2.73	2.17	AT3G02550.1	1.00E-79

In addition, to identify TFs with high GS (Gene Significance GS is the correlation between the gene and the trait) and MM (module membership MM is the correlation of the module eigengene and the gene expression profile), we performed intramodular analysis via WGCNA. A correlation coefficient (the absolute value) of more than 0.8 and P < 0.05 was used as cutoff for identifying the significance between physiological traits and modules (Fig 4B). |GS|≥ 0.8 with P < 0.05, |MM| ≥ 0.8 and P < 0.05 were used as cut-off criteria for identifying genes with high GS and MM, which were listed in S10 Table. As shown in S10 Table, 4 TFs had a high positive correlation with ABA including two MYB TFs, one ERF TF and one ZIP TF; 16 TFs were correlated with sucrose; 38 TFs were correlated with fructose (because the expression pattern of fructose was almost the same as that of glucose, these 38 TFs were also correlated with glucose); 31 TFs were correlated with quinic acid; 49 TFs were correlated with citric acid and 18 TFs were correlated with malic acid.

### Expression analysis of the candidate TFs

**Fig 6:**
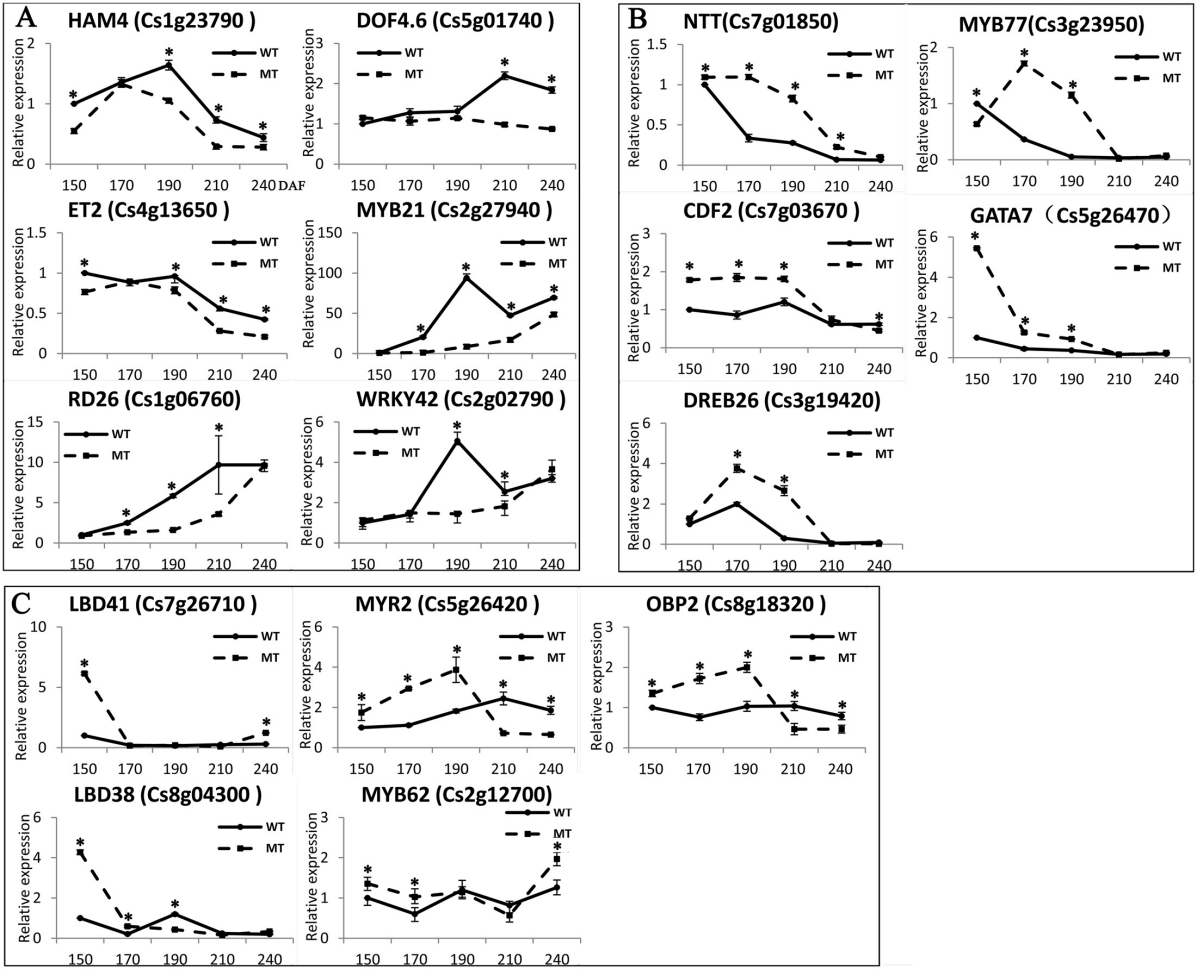
Expression analysis of TFs at five citrus fruit ripening stages. A, B and C indicate three expression patterns between MT and WT. 150, 170, 190, 210 and 240 indicate 150, 170, 190, 210 and 240 DAF, respectively. A single asterisk (*) represents a statistically significant difference (P < 0.05). Analyzed using Student's t-test.

In the present study, TFs were identified from the RNA-seq data of MT and WT at three ripening stages; therefore, we selected candidate TFs to perform expression analysis at five different ripening stages to validate the expression of TFs. Fruits harvested at 150, 170, 190, 210, and 240 DAF were selected. As expected, these 16 TFs were all differentially expressed between MT and WT. There were six TFs with up-regulated expression in WT (Fig 6A) and five TFs with up-regulated expression in MT (Fig 6B) and five TFs up/down-regulated in MT/WT (Fig 6C). For the differential ABA accumulation in WT and MT during ripening and the maturation time also delayed in MT [24], the analysis of ripening-related TFs revealed differential regulation between both cultivars.

## Discussion

Fruit ripening is a genetically programmed, highly coordinated, and irreversible phenomenon in which the physiology, biochemistry, and structure of the organ are developmentally altered to influence appearance, texture, flavor, and aroma [39]. Although the ripening phenomena varies among species, changes typically include color modification through the alteration of chlorophyll, carotenoid, and/or flavonoid accumulation; the modification of sugars, acids, and volatile profiles that affect nutritional quality, flavor, and aroma; and the modification of textural via alterations of cell wall structure and/or metabolism [3]. Transcription factors are a group of proteins that control cellular processes by regulating the expression of downstream target genes. TFs have been characterized as pivotal regulators in the ripening of different fresh fruits [4, 11, 40, 41]. In the present study, a total of 159 TFs were identified and assigned to different families. Some TFs might be significant regulators during citrus fruit ripening. The systems approach in data mining via WGCNA was particularly fruitful in identifying physiological traits, associated modules and genes for future functional studies. The hierarchical clustering analyses performed on the differentially expressed TFs between MT and WT was powerful in identifying different expression pattern TFs.

### Identification of candidate TFs involved in the formation of late-ripening trait

MT is a late-ripening mutant of WT. In the present study, 52 differentially expressed TFs between MT and WT were identified. The ERF family contained the greatest number of TFs in the DEG cluster MT vs WT (Fig 1D), indicating that the TFs of the ERF family might be key regulators for the formation of late-ripening trait of MT. The result of the GO terms and KEGG pathway enrichment analysis revealed several TFs involved in phytohormone related biological processes (S9 Table). Particularly, the TFs related to ethylene might play much more important roles. Combining the cluster analysis of gene expression, some candidate TFs, such as *MYB16* (Cs6g21530), *MYB21/77* (Cs2g27940/ Cs3g23950), *OBP2* (Cs8g18320) and *ERF4* (Cs1g07950) were screened (Table 1 and Fig 3). *MYB16* (Cs6g21530), *MYB21* (Cs2g27940) and *ERF4* (Cs1g07950) were assigned to different hormone response processes indicating that these TFs might play a wide range of regulatory roles during citrus fruit ripening (S9 Table). *ERF1* (Cs5g29870), *ARF1* (Cs5g32400) and *TGA9* (Cs8g15030) were assigned to ethylene, auxin and salicylic acid signal transduction pathways, respectively. Therefore, these TFs might be important regulators for the formation of late-ripening trait.

### Identification of candidate TFs involved in citrus fruit ripening

In this study, a total of 144 TFs, which were differentially expressed during citrus fruit ripening, were identified (S5 Table). According to the analysis of TF family distribution, the TFs of bHLH, C2H2, Dof, ERF and MYB families might play significant roles during citrus fruit ripening, particularly those of C2H2 and Dof families, which were among the top three families identified in both WT and MT. To gain a better understanding of TF roles in fruit ripening, GO-based term classification and KEGG-based pathway enrichment were performed. In the present study, some important biological processes were identified, such as ‘response to gibberellin’, ‘response to salicylic acid’ and ‘response to ethylene’ (S6 and S7 Tables). The *GAI* (Cs2g16940), a TF of GRAS family, was enriched in ‘response to gibberellin’, ‘response to salicylic acid’ and ‘response to ethylene’ and this TF was also enriched in gibberellin signal transduction pathway.

During the citrus ripening process, the ABA signal pathway may act as a central regulator, functioning in combination with other hormones, including ethylene and jasmonic acid (JA) [24, 42]. ABA is an important phytohormone involved in fruit ripening and abiotic stress [43]. In recent years, considerable progress has been made in the understanding of ABA signal transduction pathways in fruits. However, only a few TFs have been identified as important for fruit ripening, associated with the ABA response, such as *VvABF2* [4], *MYB10* [6], *MYB30* [44] and *PacMYBA* [5]. In the present study, several TFs, including *ABR1* (Cs3g21660), *RD26* (Cs1g06760), *DREB26* (Cs3g19420), *MYB77* (Cs3g23950), *MYB61* (Cs6g01750), *MYB62* (Cs2g12700), were implicated as having differential expression during citrus fruit ripening (Table 1 and S5 Table). *MYB77* and *MYB62* exhibited high correlation with the gray60 module, which was highly correlated with ABA (Figs 4 and 5). In previous studies, these TFs were shown to respond to an ABA signal involved in abiotic stress [45], lateral root growth [46] and stomatal aperture [47]. *RD26* is an activator of ABA signal transduction, and *Arabidopsis* transgenic plants overexpressing *RD26* were highly sensitive to ABA, and RD26-repressed plants were insensitive [45]. In the present study, *RD26* was up-regulated in WT during the entire ripening stage. *DREB26* can largely influence plant development, for which overexpression in *Arabidopsis* resulted in deformed plants [48]. *MYB77* could directly interact with ABA receptor *PYL8* and activate auxin signal transduction involved in lateral root growth [46]; in the present study, it was up-regulated in MT and showed a differential expression pattern in MT and WT (Fig 6). However, the functions of these TFs in the fruit ripening are unknown; therefore, these TFs may also have a similar function in response to ABA, which are valuable for further study in fresh fruit. In climacteric fruit, ERF family TFs have been implicated in hormone biosynthesis, fruit ripening and carotenoid synthesis in several fruits, such as the tomato [10, 11, 49], apple [50, 51], kiwifruit [40] and longan [52]. However, the actual functions of fruit AP2/ERF genes are still poorly understood, and furthermore, the role for these genes in nonclimacteric fruit remains unclear. In the present study, ERF family TFs were the largest cluster genes in MT vs WT (Fig 1D), suggestingthat ERF TFs may play an important role in the formation of later-ripening traits in MT and other ripening related processes. JA is another important phytohormone involved in anthocyanin accumulation [53]. Anthocyanins as one of the flavonoids are biosynthesized through the flavonoid pathway. The members of MYB-bHLH-WD40/WDR (MBW), an important regulatory mechanism for modulating anthocyanin accumulation, bHLH and MYB, have been extensively studied [17, 54, 55]. In the present study, several bHLH and MYB TFs have been identified during fruit ripening, particularly in MT. A total of 14 bHLH TFs were identified (Fig 1). Although citrus fruit do not accumulate anthocyanin, these TFs may interact with the JA signal pathway involved in the biosynthesis of flavonoids or other processes. In the present study, *MYB21* was up-regulated in WT more than 10-fold compared with MT at 190 DAF (Fig 6), which interacted with jasmonate involved in stamen filament growth in *Arabidopsis* [56]. Thus, this gene may also have other functions in citrus fruit ripening interacting with JA. In addition, *MYB21* was highly correlated with the red module, which was highly correlated with glucose and fructose (Figs 3 and 4); therefore, *MYB21* may be involved in sugar metabolism during fruit ripening. The LBD family TFs act as repressors of anthocyanin synthesis and affect additional nitrogen responses, which also regulate sectors of flavonoid biosynthesis [57].

Carbon and nitrogen metabolism, chloroplast development and the light response pathway are important for plant development and ripening. Previous studies have shown that Dof, C2H2 and GATA family TFs play pivotal roles in these metabolic pathways [19, 58–61]. Transgenic sweet potato plants overexpressing *SRF1* (a Dof TF) significantly increased the content of storage root dry matter and starch, while the glucose and fructose content drastically decreased, and the enzymes involved in sugar metabolism, and soluble acid invertase showed decreased activity in transgenic plants [58]. In wheat, the expression of *TaDof1* was influenced by the levels of nitrogen [62]. Themutation of *GNC* (GATA21) reduces chlorophyll levels and produces defects in the regulation of genes involved in sugar metabolism [63]. Plants must respond to several environmental cues, one of the most important being light. Ward et al. [20] reported that Dof TF *OBP3* was regulated by light in *Arabidopsis* thaliana and suggested a model where *OBP3* is a component in both phyB and cry1 signaling pathways, acting as positive and negative regulators, respectively. The Dof transcription factor can also respond to photoperiod regulation activating flowering. AtCDFs (CYCLING DOF FACTOR) are a group of commonly studied Dof TFs in response to the photoperiod, such as *AtCDF1* [64], *AtCDF2* [65]. C2H2 TFs function as part of a large regulatory network that senses and responds to different environmental stimuli [14]. Transgenic *Arabidopsis* plants that constitutively express *Zat12* (comprising two C2H2-type zinc finger domains) are more tolerant to high light and osmotic and oxidative stresses, and *Zat12* antisense and knockout plants are more sensitive to light, osmotic stress and salinity [66, 67]. In the present study, numerous C2H2 and Dof family TFs and several GATA family TFs were identified during citrus fruit ripening (Fig 1). Some C2H2 and Dof TFs were highly correlated with glucose, quinic acid and citric acid (S10 Table). Some TFs related to light responses, such as *GATA7* [59] and *MYR2* [68], showed differential expressed between MT and WT (Fig 6). However, the functions of these TFs in fruit development and ripening are not clear; thus, these TFs may play roles in the regulation of sugar and acid metabolism and fruit coloration responding to light.

The degradation of organic acids for fruit ripening is also important. Organic acids and soluble sugars contribute highly to the flavor and overall quality of citrus fruit. Organic acids play an essential role in energy generation, response to nutritional shortage [69] and metal ion stress [70]. Many of the structural genes involved in the metabolism have been isolated from various fruits [71–73]. In the present study, many TFs correlated with citric acid, quinic acid and malic acid during navel orange fruit ripening have been identified (S10 Table). Hence, TFs may play a significant role during the degradation of organic acids. In addition, some of the TFs correlated with organic acids were assigned to the plant hormone signal transduction pathway, such as Cs8g15030 (TGA9) and Cs6g16030 (ARF8); therefore, plant hormones may play an important role in the metabolism of organic acids.

In conclusion, in this study, we have identified numerous important TFs involved in citrus fruit ripening on the platform of the later-ripening bud mutant "Fengwan" navel orange and its wild-type "Fengjie" navel orange. The identified TFs belong to different families and are primarily assigned to the C2H2, Dof, bHLH, ERF, NAC, MYB and LBD families. Recently, several TFs have been studied in perennial fruit; herein, we determined a large cluster of TFs related to fruit ripening to provide information for the screening of TFs for further functional analysis.

## Supporting Information

S1 Fig The family distribution of transcription factors identified in all samples (A), MT (B) and WT (C). (TIF)

S2 Fig Biological process (A) and molecular function (B) enrichment analysis of the TFs differentially expressed between MT and WT during fruit ripening. Bubble color indicates the p-value; plot size indicates the frequency of the GO term in the underlying GOA database (bubbles of more general terms are larger).
(TIF)

S3 Fig Multidimensional scaling plot of dissimilarities between genes, based on topological overlap. (TIF)

S4 Fig The eigengenes expression of 32 modules clustered via WGCNA. (TIF)

S5 Fig The module assignment of TFs of WT (A), MT (B), MT vs WT (C) and the TFs included in these three clusters (D). The number of TFs in each module is indicated at the right.
(TIF)

S1 Table The values of fold-change with their respective p-values and FDR values for all genes. (XLS)

S2 Table The total genes used for WGCNA analysis. WT1, WT2 and WT3 indicate 170, 190 and 210 DAF of WT, respectively; MT1, MT2 and MT3 indicate 170, 190 and 210 DAF of MT, respectively. RPKM, reads per kb per million reads; GS, gene significance; p.GS, p value of GS; MM.module and p.MM.module indicate the correlation coefficient and P value, respectively.
(XLSX)

S3 Table Primer sequences for real-time PCR. (XLSX)

S4 Table Identified transcription factors in WT and MT. (XLSX)

S5 Table Identified differential expressed transcription factors of WT, MT and MT vs WT. WT1, WT2 and WT3 indicate 170, 190 and 210 DAF of WT, respectively; MT1, MT2 and MT3 indicate 170, 190 and 210 DAF of MT, respectively. RPKM, reads per kb per million reads; MM.module and p.MM.module indicate the correlation coefficient and P value, respectively. MT vs WT indicate the TF cluster, which is differentially expressed between MT and WT.
(XLSX)

S6 Table Gene ontology enrichment analysis (p-value < 0.01) of the TFs differentially expressed during fruit ripening in both MT and WT. (XLSX)

S7 Table Enriched (p-value < 0.01) GO term gene list differentially expressed during fruit ripening in both MT and WT relative to hormones. (XLSX)

S8 Table Gene ontology enrichment analysis (p-value < 0.01) of the TFs differentially expressed between MT and WT during fruit ripening. (XLSX)

S9 Table Enriched (p-value < 0.01) GO term gene list differentially expressed between MT and WT during fruit ripening relative to hormones. (XLSX)

S10 Table Identified TFs with high GS and MM associated with physiological traits. GS, gene significance; p.GS, p value of GS; MM, module membership; p.MM, p value of MM.
(XLS)

## Abbreviations

DAF: Days after flowering
MT vs WT: A cluster of genes differentially expressed between MT and WT
qRT-PCR: Quantitative reverse transcription-polymerase chain reaction
TF: Transcription factor
WGCNA: An R package for weighted correlation network analysis
