# Supplementary figures and images for: Genome-Wide Identification of the Transcription Factors Involved in Citrus Fruit Ripening from the Transcriptomes of a Late-Ripening Sweet Orange Mutant and Its Wild Type
Source: PLoS One. 2016 Apr 22;11(4):e0154330. doi: 10.1371/journal.pone.0154330 (PMC4841598; doi:10.1371/journal.pone.0154330)

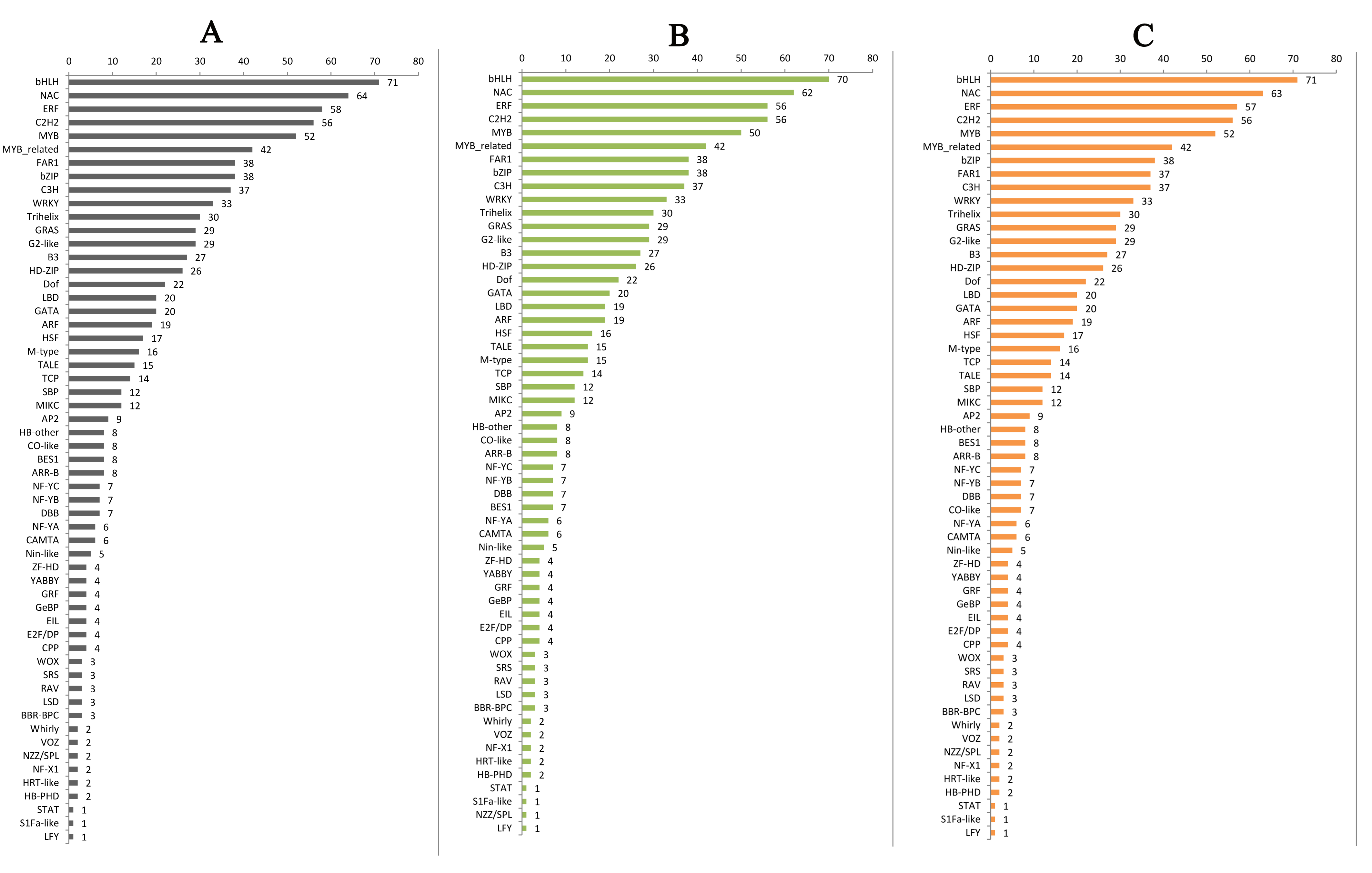

Supplement: S1 Fig — (TIF) [file pone.0154330.s001.tif]

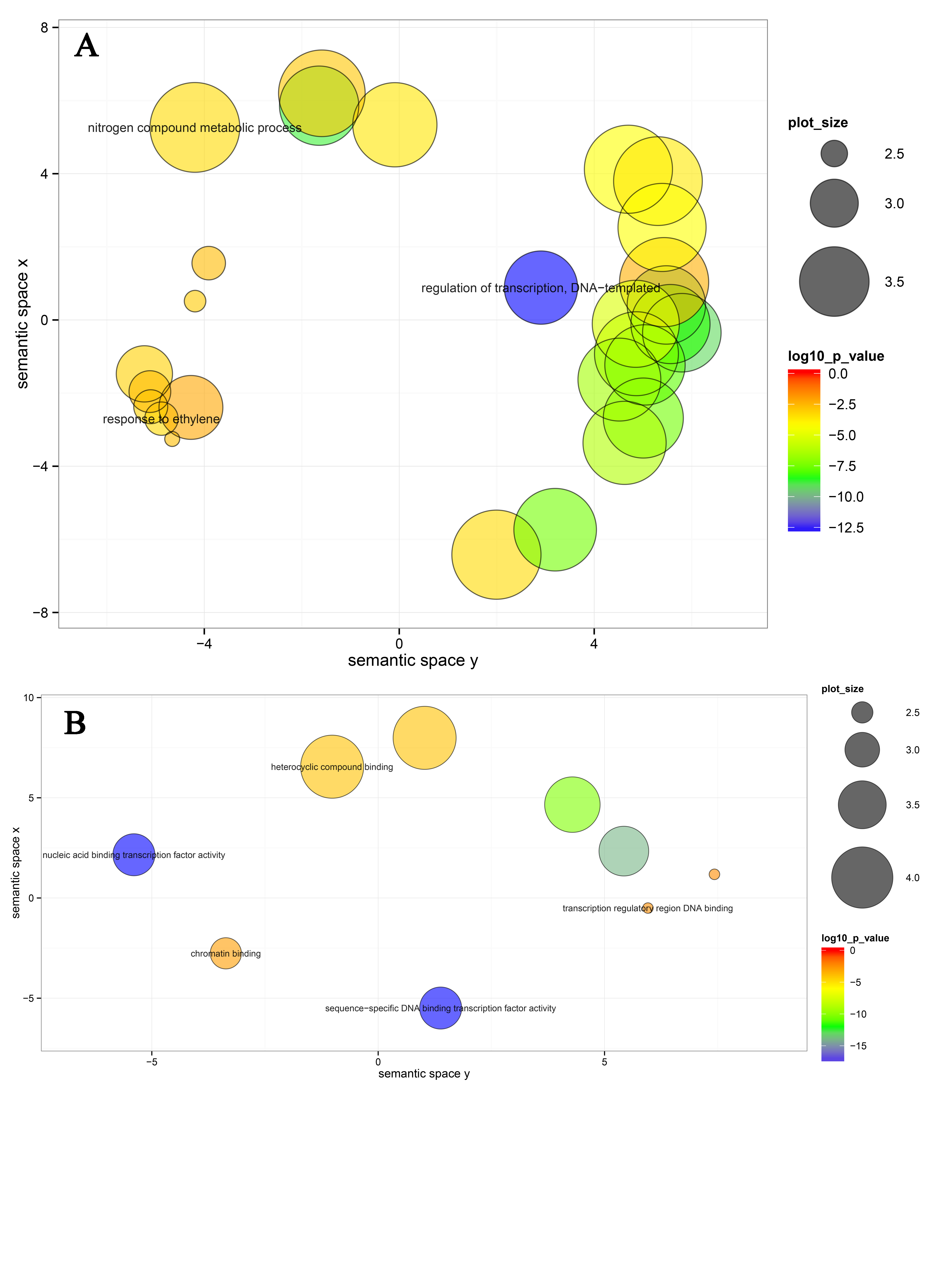

Supplement: S2 Fig — Bubble color indicates the p-value; plot size indicates the frequency of the GO term in the underlying GOA database (bubbles of more general terms are larger). (TIF) [file pone.0154330.s002.tif]

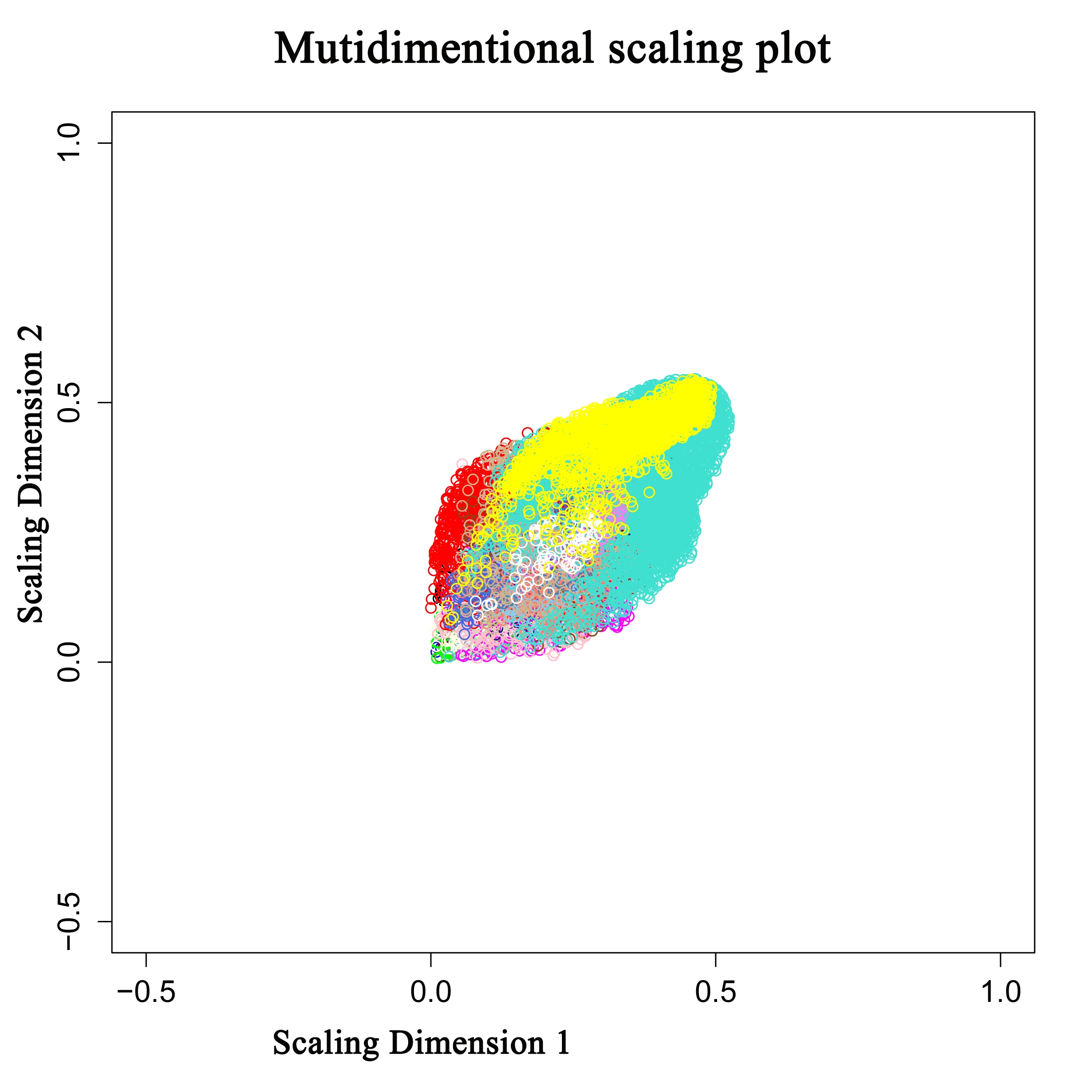

Supplement: S3 Fig — (TIF) [file pone.0154330.s003.tif]

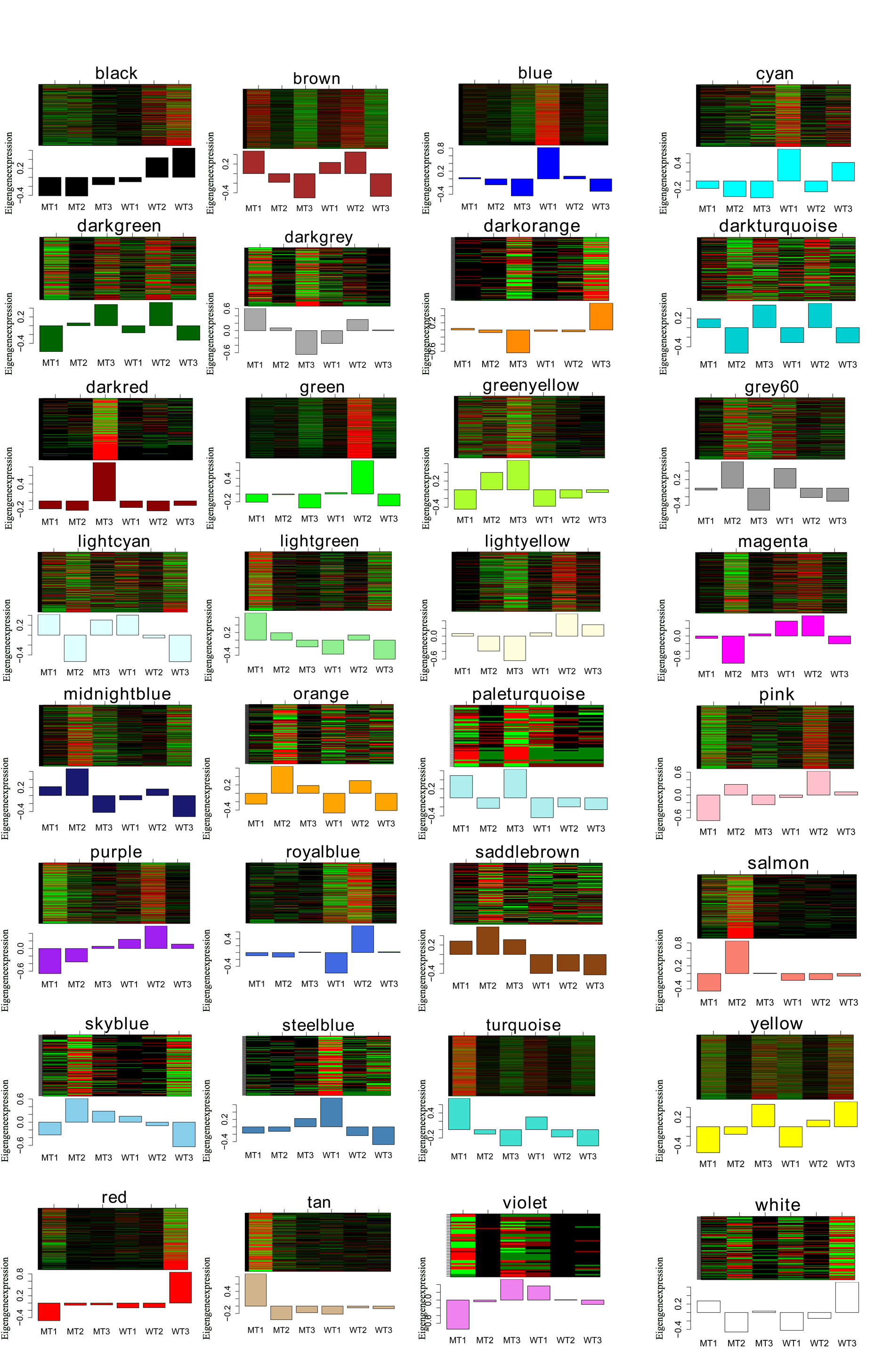

Supplement: S4 Fig — (TIF) [file pone.0154330.s004.tif]

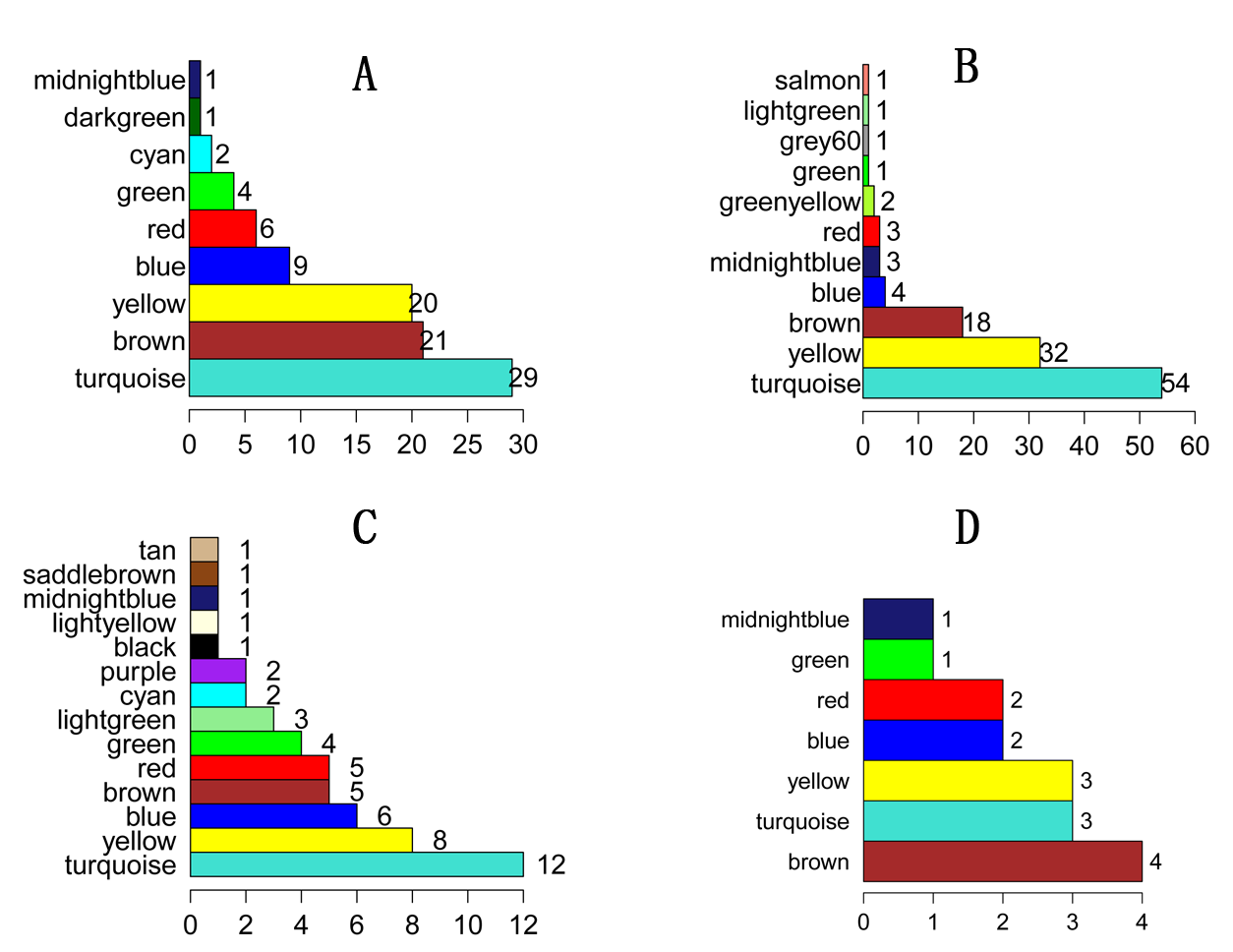

Supplement: S5 Fig — The number of TFs in each module is indicated at the right. (TIF) [file pone.0154330.s005.tif]
